# Prevalence, species composition, and associated factors of coccidiosis among domestic ruminants in Ethiopia: Systematic review and meta-analysis

**DOI:** 10.1016/j.vas.2026.100647

**Published:** 2026-04-02

**Authors:** Yihenew Getahun Ambaw, Simegnew Adugna Kallu, Assaye Wollelie Fentie, Teketay Bayleyegn Derso, Animaw Andargie Worku

**Affiliations:** a Department of Veterinary Medicine, College of agricultural sciences, Woldia University, Woldia, Ethiopia; b College of Veterinary Medicine, Haramaya University, Dire Dawa, Ethiopia

**Keywords:** Coccidiosis, Ethiopia, Meta-analysis, Ruminants, Prevalence, Eimeria species

## Abstract

Coccidiosis is a significant parasitic disease that affects the productivity and health of domestic ruminants worldwide. Although coccidiosis is one of the major causes of economic loss in ruminant farming, there is a lack of comprehensive evidence on the epidemiology of the disease in Ethiopia. Therefore, this systematic review and meta-analysis were designed to comprehensively analyze the pooled prevalence of coccidiosis and associated risk factors with identified *Eimeria* species in domestic ruminants in Ethiopia. Following the PRISMA guidelines, literature searches were conducted in PubMed, ScienceDirect, Scopus, Web of Science, and Google Scholar to retrieve articles published in Ethiopia between 2000 and 2025. This review identified a total of 31 articles; however, the number of studies increased to 40, as some articles reported multiple species. After heterogeneity assessment, the random effects model was used to estimate the pooled prevalence of coccidiosis. The estimated pooled prevalence was 42.4% (95% CI: 35.6, 49.1) among domestic ruminants in Ethiopia. In terms of species, the prevalence was higher in goats, 51.9% (95% CI: 29.1, 74.7) and sheep, 51.1% (95% CI: 35.3, 66.8), compared to cattle, 37.5% (95% CI: 30.5, 44.5). The prevalence of coccidiosis in ruminants showed a minor decreasing temporal trend over time (p = 0.061). Regarding region, the prevalence was higher in Tigray 81.8% (95% CI: 73.1, 90.6), followed by Addis Ababa 53.8% (95% CI: 32.4, 75.3), Oromia 40.6% (95% CI: 33.1, 48.0) and lower in SNNPR 20.0% (95% CI: 14.6, 25.4) with observed statistical difference (p < 0.001). *Eimeria bovis* (17.4%) and *Eimeria zurnii* (13.1%) were the most frequently identified and pathogenic *Eimeria* species in cattle. Ruminants living in the Tigray region and Addis Ababa, those with poor or medium body condition scores, studies with <384 samples and 2007–2015 published articles were significant risk factors for the prevalence of coccidiosis. This meta-analysis revealed that the prevalence of coccidiosis is high in domestic ruminants, but it is still neglected in Ethiopia, posing a significant economic impact on animal health and production. Therefore, planned preventive measures and control efforts are needed.

## 1 Introduction

Ethiopia is endowed with the largest livestock resources in Africa, with about 66 million cattle, 40 million sheep, and 51 million goats (FDRE, 2022). However, this potential is underutilized. Socioeconomic attitudes, poor management practices, limited genetic potential, inadequate nutrition, and disease challenges hinder progress (Regasa et al., 2018). Of the many diseases limiting ruminant productivity (Pal & Tariku, 2024), coccidiosis is especially significant. *Eimeria*, an intestinal protozoan parasite, causes coccidiosis in animals without zoonotic implications (Abbas et al., 2023).

Coccidiosis causes major economic losses and a serious health and production challenge, particularly in tropical and subtropical regions (Zakir et al., 2023). It reduces productivity due to poor feed conversion rate, morbidity, mortality, and both clinical and subclinical forms. High costs of prevention, control, and treatment also contribute to economic losses (Etsay et al., 2020). The global annual economic impact of coccidiosis in farm animals exceeds 3 billion dollars (Noack et al., 2019). Still, ruminant coccidiosis remains a significant cause of morbidity and mortality worldwide (Abbas et al., 2023).

*Eimeria*, an intracellular protozoan of the phylum *Apicomplexa*, is highly host-specific, infecting animals such as cattle, sheep, and goats. It has a direct life cycle and is transmitted through ingestion of sporulated oocysts from contaminated feed, water, or the environment (Regasa et al., 2018). To date, more than twelve *Eimeria* species have been identified in cattle. *E. bovis* and *E. zuernii* are highly pathogenic and cause significant mortality and morbidity (Ambaw et al., 2025). In sheep, *E. crandallis* and *E. ovinoidalis* are predominant. For goats, *E. arloingi, E. ninakohlyakimovae*, and *E. christenseni* are most frequently reported in Ethiopia (Etsay et al., 2020).

Treatments for coccidiosis include ionophores and synthetic chemicals; however, concerns about drug resistance, public health impacts, and the demand for organic products are limiting their use (Abbas et al., 2023). Despite continuous research efforts, a commercially available vaccine for ruminant coccidiosis has not yet been developed. However, herbal medicines are emerging as promising alternatives due to their diverse properties, availability, and cost-effectiveness (Abbas et al., 2023).

The first report of ruminant coccidiosis was published by Kassa et al. (1987) in Ethiopia. To prevent and control this disease, several initiatives have undertaken at the national level. These include awareness campaigns, the provision of veterinary services, enhancement of hygiene practices, drug administration, integrated control strategies, and collaborative research efforts aimed at improving disease management and prevention (Ambaw et al., 2025). According to the knowledge of researchers, all studies on ruminant coccidiosis conducted in Ethiopia were diagnosed by coprological methods (flotation, McMaster and faecal culture).

Coccidiosis has been recognized for centuries through clinical observations, historical records, necropsy findings, and standard coprological tests. However, recent advancements in diagnostic technologies have made it easier to identify *Eimeria* species in animals. Advanced immunological and molecular methods, such as PCR, immune-chromatographic tests, and nano-electrochemical biosensors, offer highly sensitive and specific diagnoses. These techniques are particularly effective for detecting *Eimeria* species that are challenging to identify and typically require a long time for faecal culture. They are accurate, sensitive, and can process multiple samples simultaneously. These tests reduce the amount of sample needed, lower material and reagent costs, save time, simplify the testing process, and make screening more efficient (Sedky et al., 2025).

Despite the country's huge livestock population and the sector's significant role in supporting economic growth and rural livelihoods, coccidiosis remains a potential challenge especially among young animals. This impact contributes to food insecurity and limit income for livestock-dependent countries like Ethiopia (Ambaw et al., 2025).

The global pooled prevalence of goat coccidiosis is 62.9%. The highest prevalence is found in North America at 92.2%, followed by Europe at 86.6%. The lowest prevalence is reported in Asia at 52.0%, with Africa showing a rate of 63.9% (Ali et al., 2025). In China, the pooled prevalence of coccidiosis is 40.0% in cattle (Li et al., 2021) and 78.7% in goats (Diao et al., 2022).

Despite these potential hazards, the distribution and risk factors of ruminant coccidiosis remain poorly understood in Ethiopia. Previous studies reported prevalence rates ranging from 12.3% in Oromia region (Elemo & Geresu, 2017) to 85.0% in Tigray region (Etsay et al., 2020), highlighting a substantial disease burden that poses risks to animal health and human food insecurity. However, these reports are dispersed, localized, and methodologically varied, hindering the development of a comprehensive national-level understanding.

Although several regional studies have documented outbreaks and risk factors in recent years, consolidate national-level evidence on the pooled prevalence and determinants of ruminant coccidiosis with the identified *Eimeria* species in Ethiopia remains limited. This gap underscores the need for further research to inform effective control strategies and animal health interventions. To address it, we conducted a systematic review and meta-analysis of studies published between 2000 and 2025. The study aimed to estimate the national pooled prevalence of coccidiosis, associated risk factors and explore the identified *Eimeria* species in domestic ruminants in Ethiopia. These findings could provide insights for coccidiosis control programs, guide veterinary health policies, and support evidence-based disease management decisions.

## 2 Materials and methods

### 2.1 Establishment of the review methodology

This systematic review and meta-analysis was structured according to the Condition (coccidiosis), Context (Ethiopia), and Population (domestic ruminants such as cattle, sheep and goat) (CoCoPop) framework (Munn et al., 2015). The study's protocol development, accomplishment, and reporting painstakingly followed the Preferred Reporting Items for Systematic Reviews and Meta-Analyses (PRISMA) Protocol 2020 guidelines (Parums, 2021). Furthermore, the protocol was officially registered with PROSPERO (International Prospective Register of Systematic Reviews), prior to commencing data collection on August 20, 2025.

### 2.2 Inclusion and exclusion criteria

This study systematically reviewed peer-reviewed, English-language cross-sectional research articles on the prevalence of coccidiosis in domestic ruminants in Ethiopia. To ensure the most current evidence, only studies published between 2000 and September 1, 2025 were included. The excluded articles were lacking sufficient information, those with missing outcomes, studies on knowledge, attitudes, and practices related to coccidiosis, personal opinions, conference proceedings, and other review articles.

### 2.3 Literature search strategy

For this systematic review and meta-analysis, data was gathered from five major academic databases: PubMed, Scopus, Google Scholar, ScienceDirect, and Web of Science. A comprehensive search was conducted using MeSH terms and keywords relevant to the study population, combined with Boolean operators OR and AND to refine the search. The initial search strategy developed for PubMed was adapted for use across the other databases. The search strategy employed a combination of terms: (Prevalence OR incidence) (Coccidiosis OR *Eimeria* OR Eimerosis) [MeSH Terms] AND (ruminants OR cattle OR sheep OR goat) [MeSH Terms] ("*Eimeria bovis*") OR ("*Eimeria zuernii*") [MeSH Terms] OR ("risk factors") OR (epidemiology) [MeSH Terms] AND (Ethiopia) [MeSH Terms]. All retrieved full-text articles were managed in EndNote version 20, with duplicates removed through manual review of titles and abstracts. Additional relevant studies were found by examining the reference lists of eligible articles, and the final literature search concluded on September 1, 2025.

### 2.4 Assessment of study quality and risk of bias

To assess the quality of the included studies, the Joanna Briggs Institute's (JBI) critical appraisal tool, specifically for prevalence studies (Munn et al., 2015), was employed. This tool has nine items, each answered with "yes," "no," "unclear," or "not applicable." The evaluation was carried out independently by two teams: Team One (TBD, AWF, and ANW) and Team Two (YGA, SAK). Discrepancies between evaluators were settled through group discussion and consultation with all researchers. For scoring, "yes" and "not applicable" answers received 1 point, while "no" or "unclear" responses (including missing or irrelevant information) scored 0 points. The total scores ranging from 0 to 9, was classified in to three groups as good (7–9), medium (4–6), or low (0–3) quality articles. Notably, all 31 articles incorporated in this review scored at least four points, indicating moderate to high quality articles.

### 2.5 Data extraction among eligible studies

After an initial relevance screen based on predefined inclusion criteria, studies proceeded to data extraction. Two independent teams (AWF, AAW and SAK) and (YGA and TBD) were extracted the data using a standardized data extraction template to collect key information, including author name, ruminant species, publication year, studies location, sample size, data collection duration, and coccidiosis prevalence. Discrepancies were resolved through team discussions. All extracted data was organized and cross-verified in Microsoft Excel (version 13) to ensure accuracy and consistency.

### 2.6 Data synthesis and statistical analysis

Full-text articles were assessed for eligibility, and relevant variables for analysis were recorded. Study results were presented in tables and forest plots. Data analysis, using the R programming language (R Core Team, 2025), involved a random-effects meta-analysis via the DerSimonian and Laird method (DerSimonian & Laird, 2015) to account for study variability. Studies involving multiple species utilize the inverse variance method for weighting. Although the DerSimonian-Laird method has limitations, particularly in cases of high heterogeneity, it remains widely employed in meta-analysis due to its simplicity and computational efficiency. This method is suitable for meta-analyses involving a moderate number of studies (> 10) and can serve as an initial analysis. It is recommended to conduct sensitivity checks with alternative methods such as REML or Hartung-Knapp (Langan et al., 2019).

A forest plot visually summarized coccidiosis distribution in domestic ruminants, including 95% confidence intervals (CI). Methodological variation between studies was quantified using the Cochrane Q test (Cochran, 1954) and the I² statistic (Higgins & Thompson, 2002); with CI estimating the true effects' range (IntHout et al., 2016).

To explore sources of variability, subgroup analyses were performed by geographic location, ruminant species, study sample size, and publication year. A sensitivity analysis, involving the sequential removal of individual studies, assessed the overall evidence's reliability. The impact of ruminant species and geographic location on variability was determined using both univariable and multivariable meta-regression models within a random-effects framework. In the meta-regression model, we categorized geographic regions based on the current Ethiopian administrative areas. Ruminant species and sampling method were treated as categorical variables. In contrast, sample size and publication year were considered continuous variables. Publication bias was evaluated through Egger's regression, and funnel plot symmetry assessments (Egger et al., 1997).

## 3 Result

### 3.1 Selection and identification of literature search results

**Fig. 1:**
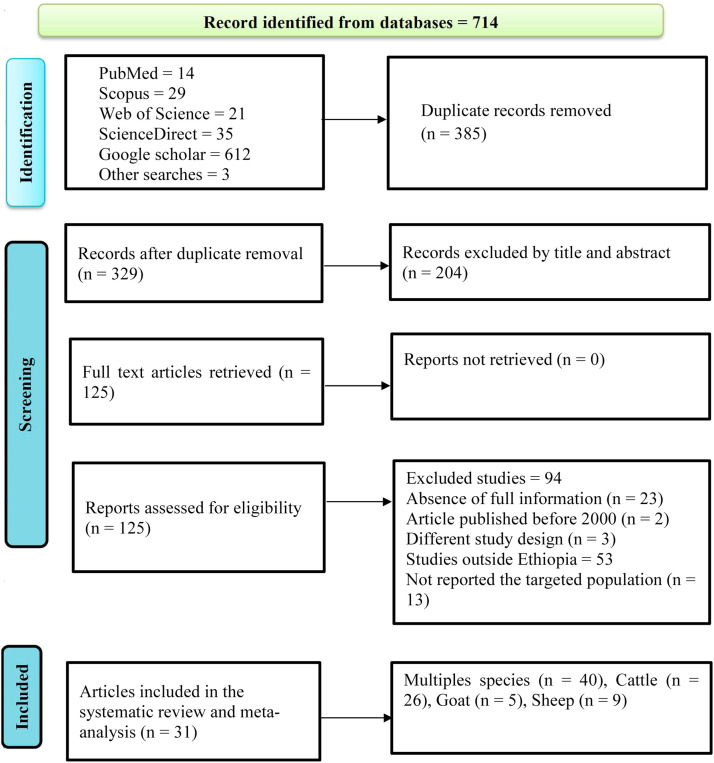
PRISMA flow diagram for the selected eligible studies in Ethiopia.

Initially, 714 studies were screened in line with the relevance of the research objectives. Subsequently, 385 articles were excluded due to duplication. Additionally, 204 studies were omitted after a detailed review of the article's title and abstract. Moreover, 94 studies were reduced due to incomplete reporting of coccidiosis, articles studied outside Ethiopia, different study designs, and published before 2000. Ultimately, 31 studies were included in the final systematic review and meta-analysis. After extracting species-specific data for every 31 articles, the number of studies increased to 40, since some articles reported results for multiple species, as illustrated in the PRISMA flow diagram (Fig. 1).

### 3.2 Description of the included studies

This review included data from 31 studies published in Ethiopia between 2000 and September 1, 2025. However, there were no published studies found between 2000 and 2006. Among the 40 species-specific datasets, the distribution by ruminant species was 26 (65.0%) for cattle, 9 (22.5%) for sheep, and 5 (12.5%) for goat. By geographical region, studies were distributed as follows: 23 (57.5%) in Oromia, 8 (20.0%) in Amhara, 4 (10.0%) in Addis Ababa, 3 (7.5%) in Tigray and 2 (5.0%) in SNNPR (Table 1, Table 2). No studies were published from Harari, Benishangul-Gumuz, Somali, Afar, or Dire Dawa administration during this period. Sample sizes ranged from 71 to 479 ruminants, with apparent prevalence varying from 12.3% in Oromia to 87.5% in Tigray (Table 1). Across all studies, the total sample comprised 11,780 ruminants, of which 4654 (39.5%) tested positive for coccidiosis.

**Table 1:** Prevalence of domestic ruminant coccidiosis and description of the included studies in Ethiopia, 2025.

Authors with years of publication	Animal species	Study regions	Number examined	Number positive	Prevalence with 95% CI	Identified *Eimeria* species
Ayana et al., 2007	Sheep	Oromia	262	175	66.8 (61.1–72.5)	Not identified
	Goat	Oromia	122	54	44.3 (35.4–53.1)	Not identified
Abebe et al., 2008	Cattle	Oromia	250	143	57.2 (51.1–63.3)	*E auburnensis, E alabamensis, E. bovis, E brasilensis, E bukidnonensis E canadensis, E cylindrica, E ellipsoidalis, E subspherica, E wyomingensis, E zurnii*
	Cattle	Addis Ababa	330	252	76.4 (71.8–80.9)	
Dawid et al., 2012	Cattle	Oromia	384	87	22.7 (18.5–26.8)	Not identified
Alemayehu et al., 2013	Cattle	Amhara	288	92	31.9 (26.6–37.3)	*E. alabamensis, E. auburnensis, E. bovis, E. ellipsoidalis, E. zuernii*
Lakew & Seyoum, 2016	Sheep	Amhara	384	88	22.9 (18.7–27.1)	Not identified
Kiltu et al., 2016	Sheep	Oromia	264	166	62.9 (57.1–68.7)	Not identified
	Goat	Oromia	120	80	66.7 (58.2–75.1)	Not identified
Hiko & Rorisa, 2016	Cattle	Oromia	384	278	72.4 (67.9–76.9)	*E. alabamensis, E. auburnensis, E. bovis, E. bukidnonesis, E. canadensis, E. cylindrica, E. ellipsoidalis, E. subspherica, E. wyomingensis, E. zuernii*
Nuriye et al., 2016	Cattle	Oromia	384	119	31.0 (26.4–35.6)	Not identified
Ayana et al., 2017	Sheep	Addis Ababa	184	110	59.8 (52.7–66.9)	Not identified
	Cattle	Addis Ababa	200	110	55.5 (48.1–61.9)	Not identified
Ayana, 2017	Cattle	Addis Ababa	334	81	24.3 (19.7–28.8)	Not identified
Elemo & Geresu, 2017	Goat	Oromia	97	16	16.5 (9.1–23.9)	Not identified
	Sheep	Oromia	479	59	12.3 (9.4–15.3)	Not identified
Teketel et al., 2017	Cattle	Oromia	384	131	34.1 (29.4 −38.9)	Not identified
Regasa et al., 2017	Cattle	Oromia	384	186	48.4 (43.4–53.4)	Not identified
Mohamed et al., 2017	Cattle	Oromia	384	122	31.8 (27.1–36.4)	*E. aubernensis, E. bovis, E. canadensis, E. cylinderica, E. ellipsoidalis, E. zuerni*
Mengistu & Malke, 2018	Cattle	Oromia	384	165	43.0 (38.0–47.9)	Not identified
Regasa et al., 2018	Cattle	Oromia	384	100	26.0 (21.7–30.4)	*E. arloingi, E. christenseni, E. ninakohyakimovae*
Gebeyehu et al., 2018	Cattle	Tigray	232	169	72.8 (67.1–78.6)	*E. bovis, E. zuernii*
Damana et al., 2018	Cattle	Oromia	266	65	24.4 (19.3–29.6)	Not identified
Worku et al., 2019	Cattle	Amhara	384	118	30.7 (26.1–35.3)	*E. bovis, E. Zurni*
Etsay et al., 2020	Sheep	Tigray	197	172	87.3 (82.7–92.0)	Not identified
	Goat	Tigray	187	159	85.0 (79.9–90.1)	Not identified
Tamrat et al., 2020	Cattle	Amhara	237	42	17.7 (12.9–22.6)	Not identified
Fayisa et al., 2020	Sheep	Amhara	313	185	59.1 (53.7–64.6)	Not identified
	Goat	Amhara	71	33	46.5 (34.9–58.1)	Not identified
Gashaw et al., 2020	Cattle	Oromia	378	200	52.9 (47.9–57.9)	Not identified
Hailu et al., 2022	Cattle	SNNPR	179	42	23.5 (17.3–29.7)	Not identified
	Cattle	Oromia	151	29	19.2 (12.9–25.5)	Not identified
Belay & Sheferaw, 2022	Sheep	Amhara	422	132	31.3 (26.9–0.357)	Not identified
Ashagrie et al., 2022	Cattle	Oromia	305	157	51.5 (45.9–57.1)	*E. alabamensis, E. auburnensis, E. bovis, E. canadinensis, E. cylindrica, E. elipsoidalis, E. subspherica E. zuernii*
Ayana et al., 2022	Cattle	Oromia	265	119	44.9 (38.9–50.9)	*E. alabamensis, E. auburnensis, E. bovis, E. ellipsoidalis, E. subspherica, E. zurnii*
	Sheep	Oromia	119	69	58.0 (49.1–66.9)	*E. ahasta, E. crandalis, E. granulosa, E. hawkinsi, E. intericata, E. ovinoidalis, E. pallida, E. parva*
Zakir et al., 2023	Cattle	Oromia	384	114	29.7 (25.1–34.3)	Not identified
Gebremeskel & Kebede, 2023	Cattle	Amhara	460	82	17.8 (14.3–21.3)	Not identified
Matheos & Edale, 2024	Cattle	SNNPR	460	82	17.8 (14.3–21.3)	*E. bovis, E. zuernii*
Ambaw et al., 2025	Cattle	Oromia	384	71	18.5 (14.6–22.4)	*E. alabamensis, E. bovis, E. ellipsoidalis, E. subspherica E. zuernii*

Note: Coprological methods, such as flotation, McMaster and faecal culture, were employed to diagnose *Eimeria* species in the relevant studies. In contrast, other studies relied solely on flotation and did not report specific *Eimeria* species. The literature search included publications from the year 2000 until September 1, 2025, and the articles were retrieved between August 20 and September 1, 2025.

**Table 2:** Subgroup analysis of coccidiosis among domestic ruminants in Ethiopia.

Groups	Categories	Included studies	Prevalence (95% CI)	Heterogeneity test (I^2^)	P value
Regions	Amhara	8	32.0 [22.1, 41.8]	96.7	<0.001
	Oromia	23	40.6 [33.1, 48.0]	97.8	
	SNNPR	2	20.0 [14.6, 25.4]	58.4	
	Tigray	3	81.8 [73.1, 90.6]	88.4	
	Addis Ababa	4	53.8 [32.4, 75.3]	98.3	
Species	Cattle	26	37.5 [30.5, 44.5]	98.2	0.182
	Sheep	9	51.1 [35.3, 66.8]	98.9	
	Goat	5	51.9 [29.1, 74.7]	97.7	
Sample size	< 384	24	50.2 [41.6, 58.8]	98.0	<0.001
	= 384	12	34.2 [25.9, 42.5]	97.6	
	> 384	4	19.7 [11.9, 27.5]	94.9	
Publication year	2007–2015	6	49.9 [33.2, 66.5]	98.2	0.043
	2016–2020	24	45.1 [36.2, 54.0]	98.7	
	2021–2025	10	31.0 [21.8, 40.2]	97.3	

### 3.3 Identified *Eimeria* species

In this review, out of 31 included studies, only11 reported the identification of *Eimeria* species in domestic ruminants. Overall, from these studies in Ethiopia, 22 species of *Eimeria* were detected in ruminants. Specifically, 14 species (*E. bovis, E zurnii, E auburnensis, E canadensis, E ellipsoidalis, E subspherica, E cylindrica, E alabamensis, E wyomingensis, E bukidnonensis, E brasilensis E. arloingi, E.ninakohyakimovae,* and *E. christenseni*) were identified in cattle, 8 in sheep (*E. ovinoidalis, E. ahasta, E. parva, E. crandalis, E. granulosa, E. hawkinsi, E. intericata,* and *E. pallida*), while no studies reported *Eimeria* species in goat. Most of the studies indicated the presence of pathogenic species, such as *E. bovis* and *E. zurnii*, in cattle (Table 1).

### 3.4 Pooled prevalence and heterogeneity among ruminant coccidiosis

**Fig. 2:**
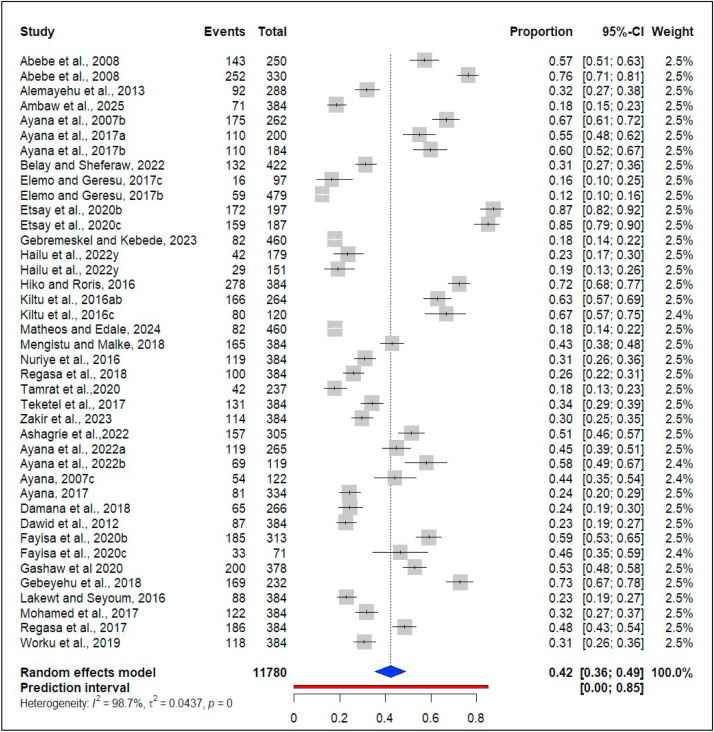
Forest plot for pooled prevalence of coccidiosis among domestic ruminant in EthiopiaNote: a = Cattle, b = Sheep, c = Goat for the presence of *Multiple species* in a single study.

The results of the meta-analysis revealed a significant level of heterogeneity across the included studies (I^2^ = 98.7%, Q _(39)_ = 2912.84, p < 0.001). As a result, a random effects model was employed to estimate the overall prevalence of ruminant coccidiosis. According to this model, the estimated prevalence of coccidiosis among ruminants was 42.4, 95% CI: [35.57; 49.05] (Fig. 2). The 95% prediction interval was [0.0; 85.13], which represents the range within which the results of a future study, based on the same population, are expected to fall (Fig. 2).

The result revealed a statistically significant heterogeneity among studies (Q _(39)_ = 2912.84, p < 0.001). The estimated variance was τ^2^ = 0.0437, and the I^2^ value was found to be 98.7%. To identify the source of heterogeneity observed in the pooled estimate from the random-effects model, sensitivity analysis, subgroup analysis, and meta-regression analysis were utilized.

### 3.5 Subgroup analysis

**Fig. 3:**
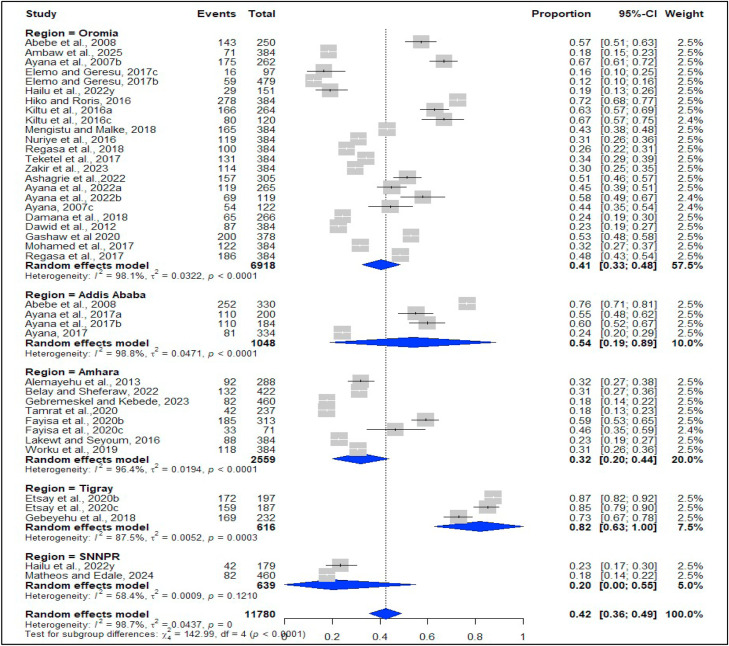
Subgroup analysis for coccidiosis based on geographic region of ruminants in Ethiopia.

A subgroup analysis was conducted based on geographical regions (Amhara, Oromia, SNNPR, Tigray, and Addis Ababa), the type of ruminants, the sampling method, the publication year, and the sample size (Table 2). Significant variations in coccidiosis prevalence were observed across the different geographic areas, sample size group and publication years. The prevalence of coccidiosis in ruminants ranged from 20.0% in SNNPR to 81.8% in the Tigray region (Table 2 and Fig. 3).

The subgroup analysis among ruminants revealed that goat had the highest infection rates at 51.9%, 95% CI: [29.1–74.7%], while cattle had the lowest rates at 37.5%, 95% CI: [30.5–44.5%] (Table 2). By sample size, the highest prevalence (50.2%, 95% CI: [41.6–58.8] was found in studies having < 384 subjects compared to studies having > 384 samples (19.7%, 95% CI: [11.9–27.5] (Table 2).

Regarding the publication year, a higher prevalence (49.9%, 95% CI: [33.2–66.5]) was found from 2007 to 2015 relative to 2021 to 2025 (31.0%, [21.8–40.2]) (Table 2).

### 3.6 Sensitivity analysis

**Fig. 4:**
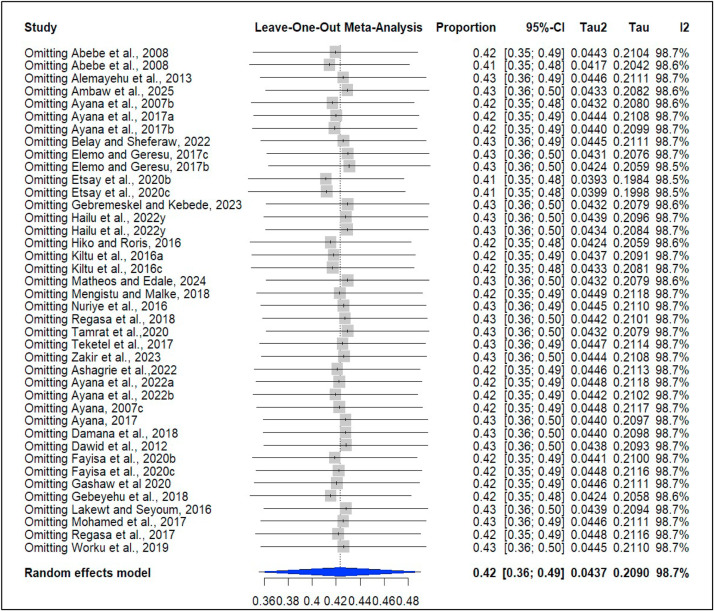
Sensitivity analysis for the prevalence of coccidiosis in domestic ruminants.

A leave-one-out sensitivity analysis was also performed to assess the influence of each study on the pooled prevalence of coccidiosis. The meta-analysis produced a pooled effect size of 42.31%, which aligned with the CIs of every individual study included. Sensitivity testing further demonstrated the stability of these findings, as removing any single study from the analysis did not meaningfully change the overall prevalence estimate (Fig. 4).

### 3.7 Influence analysis

**Fig. 5:**
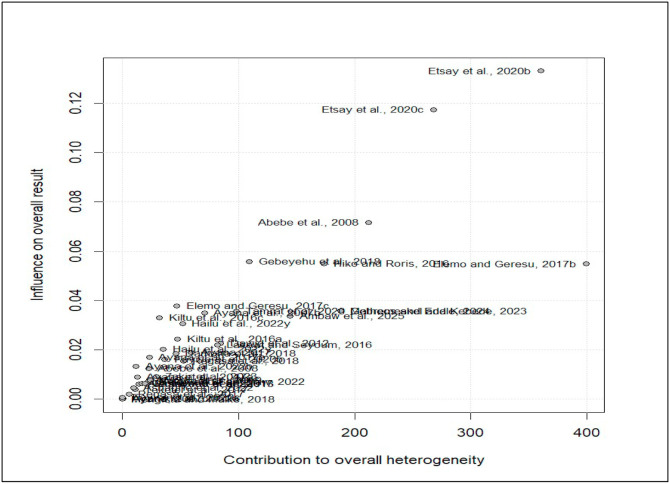
Baujat diagnostic plots for the prevalence of coccidiosis among domestic ruminants.

To identify studies that have the strongest influence on the observed heterogeneity in the current meta-analysis, the Baujat diagnostic plot was employed. Thus, among the included studies, Etsay et al. (2020b) and Etsay et al. (2020c) were found to exert a substantial influence on the overall effect size whereas Elemo and Geresu (2017c) contributes more heterogeneity in our meta-analysis (Fig. 5).

### 3.8 Meta-regression models

To identify sources of heterogeneity, we used univariable and multivariable meta-regression models. In univariable analyses, publication year and sample size were treated as continuous covariates, while study region and ruminant species were categorical variables in a mixed-effects framework. We assessed their associations with coccidiosis prevalence. Geographic region showed significant associations and explained 34.74% of heterogeneity (R^2^ = 34.74%). The multivariable model accounted for 41.83% of heterogeneity (R^2^ = 41.83%). Regression coefficients indicated that studies in Amhara (β = −0.2176), SNNPR (β = −0.3319), and Tigray (β = 0.2795) had statistical different effect sizes compared to those in Addis Ababa, after covariate adjustment (Table 3).

**Table 3:** Univariable and multivariable meta-regression analysis of heterogeneity in coccidiosis prevalence among ruminants, Ethiopia.

Moderators	Categories	R^2^	Coefficient (95% CI)	P value	R^2^	Coefficient (95% CI)	P value
Univariable meta-regression					Multivariable meta-regression		
Region	Amhara	34.74%	−0.2176 [−0.4235, −0.0117]	0.038	41.83	−0.1539 [−0.3670,−0.0593]	0.157
	Oromia		−0.1326 [−0.3147, −0.0495]	0.154		−0.0807 [−0.2615, 0.1002]	0.382
	SNNPR		−0.3319 [−0.6223, −0.0416]	0.025		−0.1957 [−0.4917, 0.1004]	0.195
	Tigray		0.2795 [0.0232, 0.5357]	0.033		0.3076 [0.0485, 0.5668]	0.020
	Addis Ababa		Reference			Reference	
Species	Cattle	5.27%	Reference			Reference	
	Sheep		0.1354 [−0.0203, 0.2911]	0.088		0.0883 [−0.0417, 0.2184]	0.183
	Goat		0.1445 [−0.0541, 0.3432]	0.154		−0.0262 [−0.2367, −0.1843]	0.807
Publication year		6.23%	−0.0137 [−0.0280, 0 0.0006]	0.061		−0.0110 [−0.0236, 0.0017]	0.090
Sample size		12.03%	−0.0007 [−0.0013, 0 0.0002]	0.013		−0.0004 [−0.0010, 0.0002]	0.195
Sampling method	Purposive	0.0%	0.1033 [−0.1159, 0.3224]	0.356			
	Random		Reference				

### 3.9 Trends on the prevalence of domestic ruminant coccidiosis

**Fig. 6:**
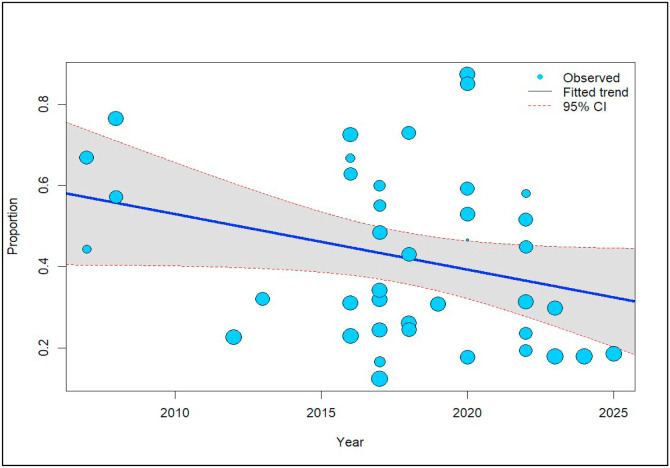
Annual trend on the prevalence of coccidiosis among domestic ruminants.

To explore the presence of temporal trends in coccidiosis prevalence among the included studies, a random-effect meta-regression model was fitted. Coccidiosis prevalence showed a non-significant declining trend (β = −0.0137, 95% CI: [−0.0280; 0.0006], p = 0.0610). This suggests that prevalence decreased by 1.37% for each additional year, on average (Fig. 6).

### 3.10 Association of sex and the prevalence of domestic ruminant coccidiosis

**Fig. 7:**
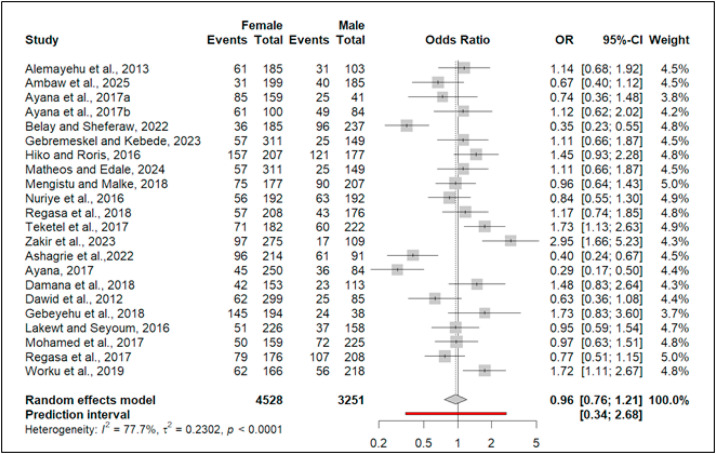
Forest plot for pooled odds ratio of sex and prevalence of coccidiosis among domestic ruminant in Ethiopia.

Of the 40 studies included in this meta-analysis, 22 reported the association between coccidiosis prevalence and ruminant sex (Fig. 7). Six studies found a significant association, while 16 did not. Pooled analysis showed no significant overall association (OR = 0.96, 95% CI: [0.76, 1.21]; Fig. 7), indicating females had 4% lower odds of coccidiosis than males.

### 3.11 Association of body condition and the prevalence of domestic ruminant coccidiosis

**Fig. 8:**
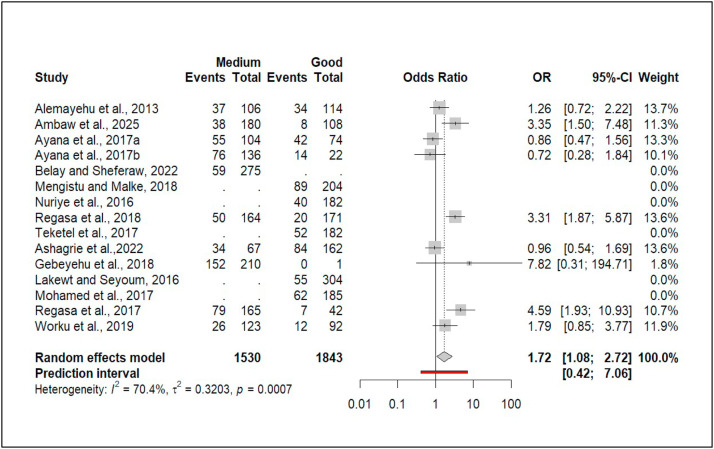
Forest plot for pooled odds ratio of medium vs good body condition and prevalence of coccidiosis among domestic ruminant in Ethiopia.

Of the 40 studies, 15 examined the association between body condition score and coccidiosis prevalence. Three reported significant differences between medium and good body condition. The pooled effect size showed ruminants in medium body condition had 72% higher odds of infection than those in good condition (OR = 1.72, 95% CI: [1.08, 2.72]; Fig. 8).

**Fig. 9:**
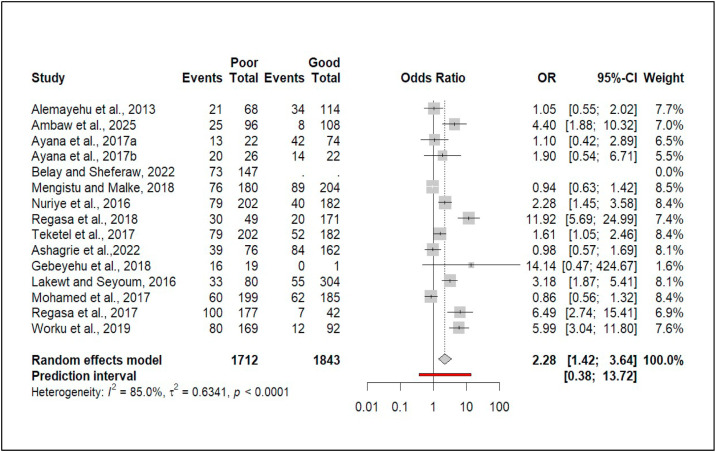
Forest plot for pooled odds ratio of poor versus good body condition and prevalence of coccidiosis among domestic ruminant in Ethiopia.

On the other hand, seven studies found a significant association between poor versus good body condition and coccidiosis prevalence (Fig. 9). The pooled odds ratio was 2.28 (95% CI: [1.42, 3.64]), indicating ruminants in poor body condition were 2.28 times more likely to be infected than those in good condition.

### 3.12 Publication bias and trim-and-fill analysis

**Fig. 10:**
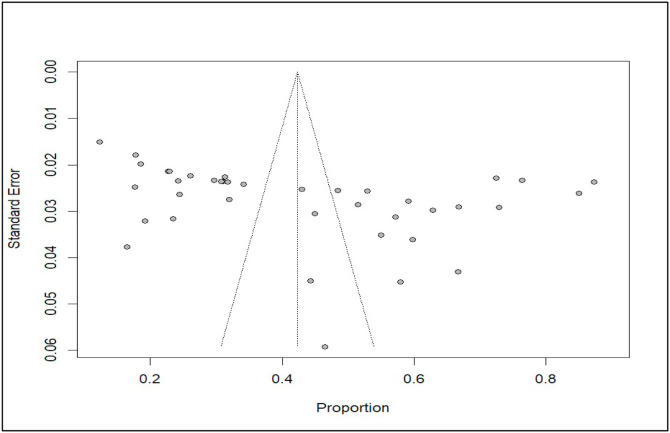
Funnel plot for the pooled prevalence of coccidiosis among domestic ruminants.

The evaluation of publication bias was employed using a combination of graphical and statistical methods. The funnel plot showed asymmetry across studies (Fig. 10). Egger's regression test confirmed this, yielding a coefficient of 15.42 (t = 3.05, p = 0.004), indicating significant publication bias or small-study effects that may influence the pooled prevalence of coccidiosis.

**Fig. 11:**
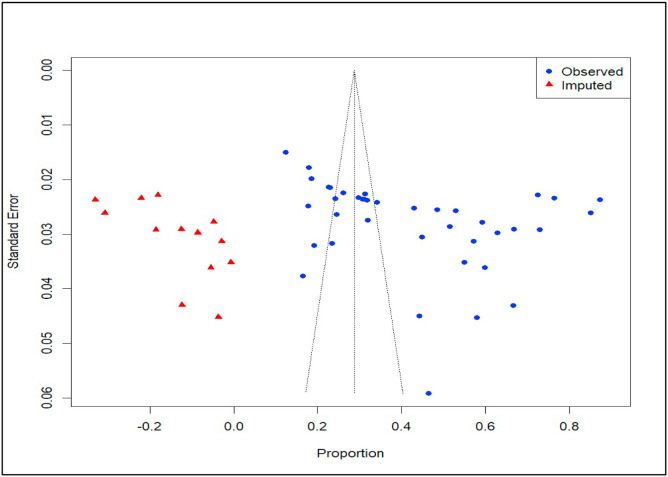
Trim and fill analysis by imputing thirteen imaginary studies.

Duval and Tweedie's trim-and-fill method was also conducted and imputed 13 studies on the left side of the funnel plot to adjust for this publication bias (Fig. 11). The adjusted pooled prevalence of ruminant coccidiosis in Ethiopia, based on 53 studies (40 observed and 13 imputed), was 28.70% (95% CI: [20.24, 37.17], with a 95% prediction intervals [0.00, 90.61].

## 4 Discussion

Coccidiosis is a significant disease affecting domestic ruminants worldwide, impacting both their health and production. It is a major parasitic disease that poses a serious global challenge to ruminant farming, adversely affecting animal well-being and productivity (Koutny et al., 2012). This often results in substantial economic losses and contributes to food insecurity. Although the economic implications of ruminant coccidiosis are not frequently studied, several reports indicate production losses, related control and prevention costs (Rodríguez-Vivas et al., 2017), particularly among young animals and in confined farming settings (Keomoungkhoun et al., 2023). The review presents consolidate evidence on the prevalence of coccidiosis and its associated risk factors among domestic ruminants, along with identified *Eimeria* species in Ethiopia. To the best of the researchers' knowledge, this report is the first systematic review and meta-analysis conducted in Ethiopia.

In Ethiopia, the pooled prevalence of coccidiosis among domestic ruminants was found to be 42.4% (95% CI: 35.57–49.05). This figure is consistent with previous reports of 42.4% in food animals across Africa (Raji & Amissah-Reynolds, 2024), 43.7% in South Africa (Matjila & Penzhorn, 2002), 38.91% in Nigeria (Ola-Fadunsin et al., 2020), 45.0% in Iran (Disfani et al., 2025), 40.0% in China (Li et al., 2021) and 47.07% in Pakistan (Rehman et al., 2011). The similarity in prevalence rates reported from different countries suggests that similar ecological and husbandry conditions, including rainfall patterns, may facilitate ongoing transmission of the disease (Keomoungkhoun et al., 2023; Makau et al., 2017).

This high ruminant coccidiosis in Ethiopia is associated with considerable economic losses, predominantly attributed to subclinical infections. These infections adversely affect feed efficiency, lead to suboptimal weight gain, increase treatment expenditures, and amplify disease susceptibility among calves and lambs. Despite a scarcity of specific quantified national figures for ruminants, existing studies underscore the significance of coccidiosis as a critical constraint on livestock productivity. Global reports indicate that coccidiosis in livestock leads to annual economic losses of >3 billion dollars (Noack et al., 2019).

To mitigate this high infection rate of coccidiosis and minimize associated economic losses in ruminants, it is imperative to implement strategies such as improved sanitation practices, rotational grazing, and the prophylactic use of anticoccidial drugs (Disfani et al., 2025).

However, the current pooled prevalence was lower than previous reports of 68.4% in Egypt (El-Alfy et al., 2020), 86.6% in Europe (Ali et al., 2025), 55.60% in Bangladesh (Chandra Deb et al., 2022), 65.4% in Indonesia (Ekawasti et al., 2021), in global meta-analysis of goats 62.9% (Ali et al., 2025), 53.61% in Mexico (Alcala-Canto et al., 2020), 91.70% in Italy (Morgoglione et al., 2020), 78.70% in China (Diao1 et al., 2022), 66.6% in Oceania (Ali et al., 2025), 83.67% at animal level and 97.97% at farm level in Austria (Koutny et al., 2012), 100% at farm level in Netherlands (Rijpert-Duvivier et al., 2021), 92.2% in North America (Ali et al., 2025) and 67.1% in Germany (Faber et al., 2002). These reports in different areas demonstrate a noticeable burden in a certain geographical regions. This variation among studies may be attributed to the use of more sensitive diagnostic tests, such as immunological (Faber et al., 2002), and polymerase chain reactions (PCR) in some reports (Keomoungkhoun et al., 2023), as well as environmental and management factors that increase ruminant exposure to infected bedding, poor hygienic conditions, water and more confined farms. More rainy seasons, coupled with poor hygienic conditions, are also considered risk factors associated with *Eimeria* infection (Keomoungkhoun et al., 2023). Copro-logical tests, such as flotation and McMaster, as most studies engaged in this analysis are known to have reduced sensitivity in detecting coccidian parasites due to uneven shedding patterns of *Eimeria* oocysts (Keomoungkhoun et al., 2023), which may partly clarify the lower pooled prevalence of ruminant coccidiosis in this report.

In addition, the increased prevalence of coccidiosis compared to previous findings may be attributed to the fact that the disease is primarily detected in young ruminants, particularly in calves and kids. This is due to adult ruminants having a higher level of protective immunity against *Eimeria* infections than younger animals. In contaminated environments, adults develop immunity through repeated exposure to oocysts (Faber et al., 2002; Keomoungkhoun et al., 2023). Furthermore, the higher prevalence of coccidiosis in developed countries such as Germany, the Netherlands, and Austria may be related to the stocking density, and diagnostic tests used to detect these protozoan parasites. The PCR technique has been extensively adopted for identifying *Eimeria* species, as it offers greater sensitivity than morphological examinations conducted under a light microscope (Faber et al., 2002; Keomoungkhoun et al., 2023).

This finding was also higher than reports from 35.0% in Thailand (Keomoungkhoun et al., 2023), 34.94% in China, 25.6% in Brazil (Cruvinel et al., 2018), 11.97% in India (Das et al., 2015), 32.8 in Kenya (Makau et al., 2017), 8.25% in Iran (Heidari et al., 2014) and 25.9% in Republic of Korea (Kim et al., 2018). The variation in ruminant coccidiosis may be attributed to differences in agricultural and farming practices among the ruminant populations in various agroecosystems (Radostits et al., 2007).

Geographic and ecological inconsistencies, along with differing study designs, could also account for the differences in disease occurrence observed in this study compared to previous research. For instance, some study areas may experience higher rainfall than others, and many ruminants in these regions might be managed under suboptimal farming systems, often grazing in swampy pastures. This environment could contribute to increased cases of ruminant coccidiosis in certain locations (Chandra Deb et al., 2022). Additionally, variations in the incidence of ruminant coccidiosis might stem from several factors, including the presence of co-infecting pathogens, the number of oocysts ingested by the ruminants, climatic conditions, seasonal changes, management practices, the immune status of young ruminants, the parasite control plan, and the diagnostic methods utilized in various studies (Rehman et al., 2011).

In Ethiopia, the prevalence of ruminant coccidiosis varies significantly across different geographical regions, publication years, and sample sizes. The subgroup analysis demonstrated that the prevalence of coccidiosis was highest in the Tigray region, followed by Addis Ababa and Oromia. This variation can be attributed to several risk factors. Geographical regions influence the risk of ruminant coccidiosis primarily through their effects on climate, including temperature and humidity, as well as the types of livestock management systems in place. These factors affect the survival and sporulation of *Eimeria* oocysts in the environment. Conditions such as rapid growth, contaminated feed and water, and poor ventilation can further increase vulnerability to this disease (Ekawasti et al., 2021; Makau et al., 2017).

On the other hand, a significant positive correlation exists between infection risk and rising temperatures. Warm environments favor the proliferation of *Eimeria* parasites, as both schizogony and sporogony occur in warm, humid locations with sufficient oxygen. Small temperature changes influence the development rate of oocysts and the duration of their survival in the environment, which in turn affects infection rates in animals (Makau et al., 2017).

In terms of publication year, a higher prevalence of ruminant coccidiosis was observed in articles published between 2021–2025 and 2016–2020 compared to those from 2000–2015. The trend analysis also indicates a gradual decline in the prevalence rate of ruminant coccidiosis in recent years, although this trend is not statistically significant. This decline may be attributed to the implementation of various initiatives at the national level aimed at combating diarrheal diseases, including coccidiosis in animals. These initiatives include awareness campaigns, improved veterinary service coverage, mass drug administration, integrated control strategies, and research collaborations (Thrusfield, 2018). This may improve the community knowledge, disease control practice, and the level of veterinary cares provided for animals.

On the other hand, this rise in prevalence over time could be *Eimeria* parasites develop resistance rapidly following the introduction of drugs in the field. Resistance has been reported for almost all currently used drugs. The current approach to delay the onset of resistance is to employ rotation programs combined with good husbandry, chemoprophylaxis or live parasite vaccination. In view of advances in biotechnology, modern approaches towards the discovery of novel resistance breaking drug candidates may be anticipated. However, for effective prevention and control of coccidiosis in livestock in the future, both novel cost-effective drugs with unique modes of action for preventative chemotherapy and subunit or recombinant vaccines are urgently needed or discovered (Noack et al., 2019).

Regarding sample size, studies with fewer than 384 samples typically reported a higher prevalence of coccidiosis, followed by studies with exactly 384 samples and those with >384 samples. The increased prevalence in smaller sample sizes may be sampling bias or specific high-risk local factors, rather than the sample size itself and the true biological effect, causing the disease. Larger and more representative samples tend to average out these local variations, resulting in a more accurate overall estimate of coccidiosis prevalence (Naing et al., 2022; Thrusfield, 2018). Smaller samples are more prone to bias, potentially misrepresenting the larger population and resulting in inflated disease prevalence. If a sample disproportionately includes individuals from high-risk areas, it may reflect localized factors such as poor management, overcrowding, or favorable environmental conditions for coccidiosis. The limited number of cases increases the risk of random variation skewing results, allowing a few cases to unduly influence the overall prevalence rate. In contrast, larger samples mitigate these variations, providing a more accurate estimate of coccidiosis prevalence across diverse conditions (Naing et al., 2022; Thrusfield, 2018).

The body condition of ruminants is significantly linked to coccidiosis. Ruminants with poor body condition are at a higher risk of coccidiosis infection compared to those in better condition. This finding aligns with previous researches (Ambaw et al., 2025; Regasa et al., 2018; Worku et al., 2019). However, it contradicts other studies (Ashagrie et al., 2022; Gebeyehu et al., 2018; Rehman et al., 2011), which may be due to the fact that body condition directly affects overall health and is often considered a key factor in assessing fitness (Peig & Green, 2009).

Even though all ruminants have similar access to *Eimeria* oocysts, the higher prevalence of calf coccidiosis in animals with poor body condition may be attributed to the greater immunity found in those in better physical condition. Additionally, inadequate body condition can result from insufficient feeding or nutritional management, which may weaken the immune systems of ruminants, reduce their resistance to infections, and consequently increase the prevalence of illness among those in poor condition (Radostits et al., 2007).

The current review discloses that a total of 22 species of *Eimeria* have been identified in Ethiopian ruminants. When examining the detected *Eimeria* species by type of ruminant, we found 14 species in cattle, 8 species in sheep, and authors have not identified species in goats. There are around 8 species of *Eimeria* identified in small ruminants; among these, a few are potentially highly pathogenic, while several others have minimal pathogenic effects under normal conditions. That is why most *Eimeria* infections are not severe in ruminants (Etsay et al., 2020). The finding was similar to previous reports in African food animals (Raji & Amissah-Reynolds, 2024). In cattle, *E. bovis* and *E. zuernii* were the most pathogenic and most frequently reported *Eimeria* species. The result was in agreement with (Keomoungkhoun et al., 2023; Raji & Amissah-Reynolds, 2024). In this report, all studies focused on ruminant species, with cattle leading, followed by sheep and goats. This trend may be due to the significant economic impact of *Eimeria* infections on the dairy sector in Ethiopia, which has attracted more attention from researchers and funding organizations. Support for this idea comes from studies indicating that coccidiosis is a contributing factor to diarrhea, production losses, and even morbidity and mortality, particularly among calves aged three weeks to six months (Rodríguez-Vivas et al., 2017).

In general, the findings of this study highlight the widespread prevalence of *Eimeria* infections among ruminants in Ethiopia, underscoring an urgent need for targeted control measures. The notably high prevalence of coccidiosis in goats, particularly among ruminants with poor to moderate body condition scores and those located in regions such as Tigray and Addis Ababa raises significant concerns regarding the health and productivity of these animals, which are vital to the livelihoods of several poor communities.

Furthermore, future researches should embrace a One Health framework, which includes evaluation of environmental factors, host immunity, and management practices that influence the transmission dynamics of *Eimeria* species among Ethiopian domestic ruminants. The incorporation of PCR-based diagnostic technologies with DNA sequencing will enable species-level identification, facilitating differentiation among morphologically similar *Eimeria* species and providing clarity on mixed infections. Additionally, molecular epidemiology can disclose genetic diversity among ruminants in Ethiopia and investigate potential drug resistance for different *Eimeria* species (Disfani et al., 2025). Moreover, the spatial and temporal dynamics of coccidiosis in relation to climate change, such as variations in temperature, humidity, and grazing practices, remain inadequately explored. Thus, prospective follow-up studies are essential to discover current and future level of *Eimeria* species resistance for anticoccidial drugs, seasonal infection trends, reinfection rates, and persistence within individuals and herds among Ethiopian ruminants.

## 5 Limitation of the study

This meta-analysis encountered with barriers related to diagnostic consistency, homogeneity of research design and geographic coverage. Some areas of the country were underrepresented, which may influence the national applicability of the findings. Most studies were conducted in cattle compared to sheep and goats, which may bias the generalizability of the findings. Bias may also introduced due to excluding studies published in non-English languages. High heterogeneity may have impact on the generalizability of pooled estimate. Many studies fail to identify *Eimeria* species across the country, resulting in an underrepresentation of the detected *Eimeria* species for generalization. Furthermore, application of different diagnostic techniques in different studies may result in difference in accuracy of prevalence estimates, and the popularity of cross-sectional design limits our ability to comprehend long-term disease dynamics and seasonal variations.

## 6 Conclusion

Coccidiosis remains a common and economically significant protozoan disease affecting domestic ruminants in Ethiopia. The included research articles show considerable variability in prevalence rates, which can be largely attributed to factors such as geographic location, sample size, and the years of publication. Higher prevalence rates of coccidiosis were found in studies with smaller sample sizes, older research, and in ruminants with poor body condition. Living in the Tigray region and having poor and medium body condition scores are significant risk factors for the prevalence of ruminant coccidiosis. Various *Eimeria* species have been identified in ruminants, with the most pathogenic species, such as *E. bovis* and *E. zuernii*, being predominant in nearly all studies reporting on *Eimeria* species. These results highlight the necessity for better animal management practices, region-specific control measures, and enhanced surveillance systems. To improve the precision of prevalence estimates and support evidence-based policy decisions, future studies should focus on filling in the gaps in data quality and reporting, especially through standardized diagnostic techniques and study designs. Future researchers should also focus on assessing the economic impact of coccidiosis and evaluating the level of anticoccidal drug resistance of *Eimeria* species among ruminants in Ethiopia.

## Data sharing statement

The data used for analysis are available from the corresponding author upon reasonable request.

## Funding

There is no specific funding for this work.

## Ethical approval

Not applicable for this systematic review and meta-analysis.

## Declaration of generative AI and AI-assisted technologies in the writing process

During the preparation of this manuscript, the authors used Grammarly and Perplexity to enhance readability and language clarity. After using this tool/service, the authors reviewed and edited the content as needed and take full responsibility for the content of the published article.

## CRediT authorship contribution statement

**Yihenew Getahun Ambaw:** Writing – original draft, Methodology, Investigation, Formal analysis, Data curation, Conceptualization. **Simegnew Adugna Kallu:** Writing – review & editing, Methodology, Formal analysis. **Assaye Wollelie Fentie:** Writing – review & editing, Methodology, Investigation, Formal analysis. **Teketay Bayleyegn Derso:** Writing – review & editing, Visualization, Methodology. **Animaw Andargie Worku:** Writing – original draft, Methodology, Investigation, Formal analysis, Conceptualization.

## Declaration of competing interest

The authors declare that they have no known competing financial interests or personal relationships that could have appeared to influence the work reported in this paper.
